# Death Cafés for prevention of burnout in intensive care unit employees: study protocol for a randomized controlled trial (STOPTHEBURN)

**DOI:** 10.1186/s13063-020-04929-4

**Published:** 2020-12-11

**Authors:** Marjorie E. Bateman, Rachel Hammer, Abigail Byrne, Nithya Ravindran, Jennifer Chiurco, Sasha Lasky, Rebecca Denson, Margo Brown, Leann Myers, Yuanhao Zu, Joshua L. Denson

**Affiliations:** 1 Department of Medicine, Tulane University School of Medicine, 1430 Tulane Avenue, New Orleans, LA 70112 USA; 2 Department of Psychiatry, Tulane University School of Medicine, New Orleans, LA USA; 3 Department of Biostatistics and Data Science, Tulane University School of Public Health and Tropical Medicine, New Orleans, LA USA; 4 Section of Pulmonary Diseases, Critical Care & Environmental Medicine, Tulane University School of Medicine, New Orleans, LA USA

**Keywords:** Burnout, Anxiety, Depression, Critical care, Healthcare workers, Occupational stress, Moral distress, Behavioral symptoms, Work place retention, Death Café, Teledebriefing, Virtual debriefing, Randomized controlled trial

## Abstract

**Background:**

Burnout is an occupational syndrome that leads to mental health problems, job turnover, and patient safety events. Those caring for critically ill patients are especially susceptible due to high patient mortality, long hours, and regular encounters with trauma and ethical issues. Interventions to prevent burnout in this population are needed. Preliminary studies suggest debriefing sessions may reduce burnout. This study aims to assess whether participation in regular debriefing can prevent burnout in intensive care unit (ICU) clinicians.

**Methods:**

A randomized controlled trial will be conducted in two large academic medical centers. Two hundred ICU clinicians will be recruited with target enrollment of 100 physicians and 100 non-physicians (nurses, pharmacists, therapists). Participants must have worked in the ICU for the equivalent of at least 1 full time work week in the preceding 4 weeks. Enrolled subjects will be randomized to virtually attend biweekly debriefing sessions facilitated by a psychotherapist for 3 months or to a control arm without sessions. Our debriefs are modeled after Death Cafés, which are informal discussions focusing on death, dying, loss, grief, and illness. These sessions allow for reflection on distressing events and offer community and collaboration among hospital employees outside of work.

The primary outcome is clinician burnout as measured by the Maslach Burnout Inventory (MBI) Score. Secondary outcomes include depression and anxiety, as measured by the Patient Health Questionnaire 8 (PHQ-8) and Generalized Anxiety Disorder 7-item scale (GAD-7), respectively. Questionnaires will be administered prior to the intervention, at 1 month, at 3 months, and at 6 months after enrollment. These values will be compared between groups temporally. Qualitative feedback will also be collected and analyzed.

**Discussion:**

With ICU clinician burnout rates exceeding 50%, Death Café debriefing sessions may prove to be an effective tool to avert this debilitating syndrome. With COVID-19 limiting social interactions and overloading ICUs worldwide, the virtual administration of the Death Café for ICU clinicians provides an innovative strategy to potentially mitigate burnout in this vulnerable population.

**Trial registration:**

ClinicalTrials.gov NCT04347811. Registered on 15 April 2020

## Administrative information

The order of the items has been modified to group similar items (see http://www.equator-network.org/reporting-guidelines/spirit-2013-statement-defining-standard-protocol-items-for-clinical-trials/).

**Table:** 

**Title {1}**	**Death Caf**é**s for prevention of burnout in intensive care unit employees: study protocol for a randomized controlled trial (STOPTHEBURN)**
**Trial registration {2a and 2b}.**	ClinicalTrials.gov, NCT04347811. First posted April 15, 2020.
**Protocol version {3}**	5/4/2019, Version 3
**Funding {4}**	The Spirit of Charity Foundation and Tulane University School of Medicine served as sources of funding for the research reported.
**Author details {5a}**	^1^Department of Medicine, Tulane University School of Medicine, New Orleans, LA ^2^Department of Psychiatry, Tulane University School of Medicine, New Orleans, LA ^3^Department of Biostatistics and Data Science, Tulane University School of Public Health & Tropical Medicine, New Orleans, LA ^4^Section of Pulmonary Diseases, Critical Care & Environmental Medicine, Tulane University School of Medicine, New Orleans, LA
**Name and contact information for the trial sponsor {5b}**	Tulane University School of Medicine
**Role of sponsor {5c}**	The sponsor and funding body had no role in the design of the study or the collection, analysis, and interpretation of the data and writing the manuscript.

## Introduction

### Background and rationale {6a}

Burnout is an occupational syndrome characterized by emotional exhaustion, distant or indifferent attitude toward work, and reduced sense of personal accomplishment [1]. It affects physicians and ancillary healthcare providers on a national scale [2–7]. The prevalence of burnout is as high as 60% in hospital employees at one of our institutions, an urban, academic, Level One trauma center [7]. Those caring for critically ill patients are especially susceptible to burnout due to features specific to the intensive care unit (ICU) such as high patient morbidity and mortality, long hours, and regular encounters with trauma and ethical issues. These experiences lead to moral distress and compassion fatigue [5, 8–12].

Burnout not only impacts the ability to enjoy work but also may lead to depression, posttraumatic stress disorder, substance abuse disorder, and suicidality [4, 5, 13, 14]. It can increase the rate of job turnover, leading to caregiver shortages [5, 10]. Most significantly, burnout is known to contribute to patient safety events [5, 15]. Physicians and nurses with burnout are more likely to make medical errors, deliver a lower quality of care, and communicate poorly with their patients [4, 14, 16–28]. Burnout is associated with higher 30-day mortality and rates of hospital-acquired infections [5].

In spite of the growing awareness of the consequences of burnout, interventions to prevent or address burnout are sparse in the literature [5, 15, 29]. Most interventions to date have focused on reduction in work hours, development of wellness curricula, and mindfulness promotion [29–32]. Preliminary studies suggest that debriefing opportunities may reduce burnout through enhancement of social support and interprofessional collaboration [33–37].

Death Cafés are a specific form of debriefing which have emerged internationally for the general public using informal discussion on topics of death, dying, loss, and illness to mitigate distress [38, 39]. During the COVID-19 pandemic, many community-based Death Cafés shifted to virtual modalities to respect social distancing guidelines. Healthcare worker specific Death Cafés have been described with similar structure and function to community initiatives with the aim to foster reflection on distressing patient events while developing a sense of community and collaboration among employees [7]. This would be the first study to report on the effects of hospital-based Death Cafés and the first study to report on the efficacy of virtual debriefing sessions for burnout prevention in an ICU setting.

### Objectives {7}

Systematic Trial of PrevenTing Healthcare Employee Burnout Using Reflection & Nourishment (STOPTHEBURN) is a single-center 2-arm randomized controlled trial that will evaluate the impact of Death Café debriefing interventions on burnout in healthcare employees. We hypothesize that participation in Death Cafés will lead to lower rates of burnout in physicians and staff (nurses, pharmacists, therapists).

### Trial design {8}

STOPTHEBURN is a parallel group randomized controlled trial with a 1:1 allocation ratio.

## Methods: participants, interventions, and outcomes

### Study setting {9}

Our two study sites are academic hospitals with large medical and surgical ICUs in New Orleans, LA. Notably, one of the hospitals has a wellness taskforce, B-Well, with volunteer staff participation to address professional burnout through supporting employee health and wellbeing. Since 2017, B-Well sponsors quarterly Death Cafés for hospital employees facilitated by authors RH and NR. Participation in the B-Well events is voluntary and not specific to ICU clinicians.

### Eligibility criteria {10}

Participants will be enrolled biweekly and are eligible if they are physicians (residents, fellows, and attendings), nurses, pharmacists, or therapists (respiratory, physical, speech) who have worked the equivalent of at least 1 full time work week (for their respective profession) in the ICU over the prior 4 weeks.

### Who will take informed consent? {26a}

Study investigators will obtain informed consent from potential trial participants. This will be done in person for participants recruited in person and electronically for participants recruited via e-mail. Study investigators will provide potential participants with information about the study, including the procedures involved for both arms of the study, risks of participation, and potential benefits of participation. Written informed consent will be obtained for subjects recruited in person. For subjects recruited via e-mail, online submission of the consent form will constitute informed consent.

### Additional consent provisions for collection and use of participant data and biological specimens {26b}

This trial does not involve collecting biological specimens for storage.

## Assignment of interventions: allocation

### Sequence generation {16a}

Randomization will utilize an online random number generator and will be performed in pairs such that each time a physician is randomized to a study arm, a non-physician will also be randomized to that study arm for balance.

### Concealment mechanism {16b}

Each physician and non-physician name will be blindly placed into an envelope that contains a computer-generated random number.

### Implementation {16c}

The allocation sequence is generated by the computer, and the concealment is performed by a member of the research team. After study investigators open the sealed envelope, they can enroll the participants and assign them to interventions.

## Assignment of interventions: blinding

### Who will be blinded {17a}

Trial participants will be notified of their randomly allocated condition (Death Café intervention group or control group) and the associated study procedures. The psychotherapists hosting the Death Cafés cannot be blinded to group allocation as they will be leading the intervention. Members of the research team will not be blinded to the assigned arm of the study if they are directly assisting with coordination of the Death Café debriefing sessions. Other members of the study team who are not hosting or coordinating these sessions will be blinded to each participant’s group allocation. An independent statistician will conduct data analysis and will be blinded to the nature of each group undergoing analysis (will be labeled as “A” and “B”).

### Procedure for unblinding if needed {17b}

Unblinding will not be performed unless requested by the Tulane University Institutional Review Board, which is the regulatory committee that approved this study.

## Interventions

### Explanation for the choice of comparators {6b}

In order to test the study hypothesis, psychometric outcomes of those who participate in Death Café debriefing interventions will be compared with those not participating in debriefing.

### Intervention description {11a}

In the intervention arm, participants will be asked to participate in at least 4 Death Café debriefing sessions over 3 months hosted by one of our two psychotherapists (RH and NR) [7]. These sessions will occur virtually through a teleconferencing platform in the early evening (after daytime work hours) in an effort to optimize attendance. Subjects will be notified of the date, time, location, and what to expect in the session in advance via the contact information provided on their consent form.

### Criteria for discontinuing or modifying allocated interventions {11b}

Participants may request to discontinue participation in the study at any time.

### Strategies to improve adherence to interventions {11c}

RH and NR will lead the Death Café debriefing sessions virtually in a manner consistent with previous work [7]. This format, as well as the full study protocol, will be reviewed quarterly at investigator meetings. Death Café debriefing sessions will be randomly attended by other study investigators throughout the course of the study to monitor consistency of adherence to the Death Café intervention structure.

### Relevant concomitant care permitted or prohibited during the trial {11d}

Participants will be permitted to engage in concomitant care as each deems personally necessary for burnout, depression, or anxiety symptoms during the trial.

### Provisions for post-trial care {30}

If subjects experience distress, they can contact the trained psychotherapists on our study team (RH and NR) for triage to appropriate mental health care. Their contact information will be provided to subjects in the consent document. For acute suicidal emergencies, participants will consent to a plan to call 9-1-1 or present to the nearest emergency room.

### Outcomes {12}

The primary outcome of the study is employee burnout as measured by the Maslach Burnout Inventory (MBI) Score*.* Mean total MBI score and mean subscores will be calculated. Presence of burnout is defined by high values of depersonalization (> 10/30) and emotional exhaustion (> 27/54) with low values for personal accomplishment (< 33/48) as defined in previous studies [40].

Secondary outcomes include employee depression and employee anxiety. These will be determined by the Patient Health Questionnaire 8 (PHQ-8) and Generalized Anxiety Disorder 7-item scale (GAD-7) which evaluate frequency of symptoms over the last 2 weeks. Scores of 10 or higher on these assessments are considered to indicate clinically significant depression or anxiety, respectively [41–43].

Scores will be compared between groups and analyzed from the beginning to the end of the intervention period (at enrollment, 1 month, 3 months, and 6 months after enrollment). The surveys will also include free text response options allowing participants in the intervention arm to comment on strengths and areas for improvement of the intervention.

### Participant timeline {13}

**Fig. 1:**
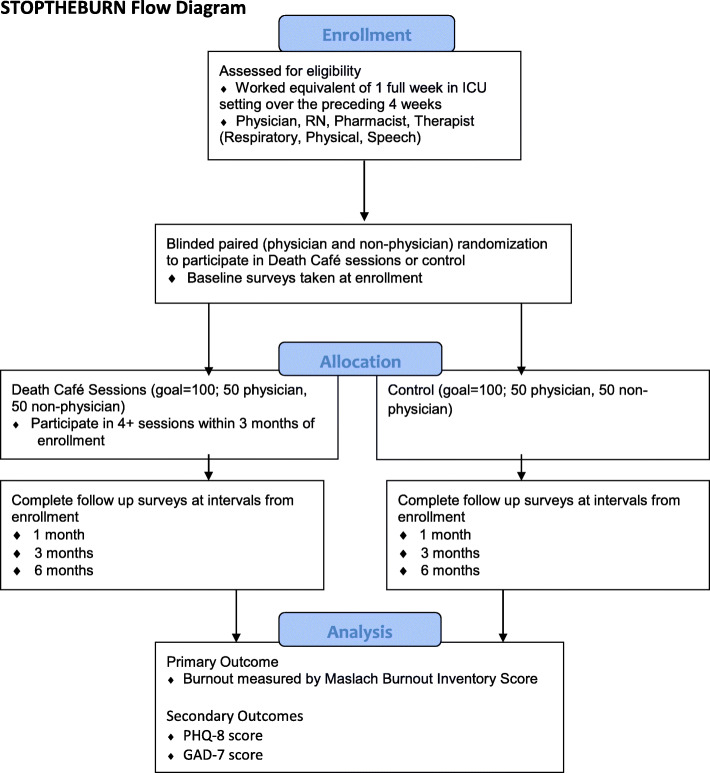
STOPTHEBURN flow diagram

**Fig. 2:**
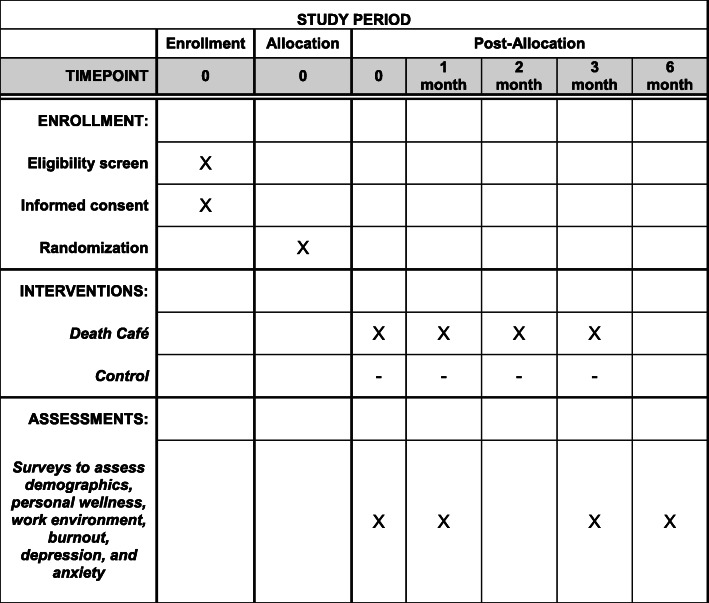
SPIRIT diagram depicting the timeline of the STOPTHEBURN study

Subjects in the Death Café arm of the study will participate in 4 debriefing sessions over 3 months from the time of enrollment. Surveys will be administered by email to participants in both arms of the study prior to the intervention, at 1 month, at 3 months, and at 6 months after enrollment. Figures 1 and 2 demonstrate the flow of participants through the study.

### Sample size {14}

The target enrollment is 200 ICU employees, of which we expect approximately 100 physicians and 100 non-physicians (nurses and therapists) in the study. This is based on a power analysis we performed for the ability to detect 20% reduction in burnout prevalence or a change of greater than 4 in mean Maslach Burnout Inventory Score (power of 0.8, alpha of 0.05).

### Recruitment {15}

Recruitment will be performed in person at each ICU site and electronically via email to employee listservs on a biweekly basis until target enrollment is reached. Recruitment will involve screening for eligibility, providing potential participants with information about the study, and obtaining informed consent. Participants will be offered compensation for completion of a minimum number of study procedures. This will be $140 for completion of the Death Café study arm and $40 for the control arm.

## Data collection and management

### Plans for assessment and collection of outcomes {18a}

Relevant information related to the outcome measures is noted under the outcomes section. Data will be collected via surveys generated using Research Electronic Data Capture (REDCap) software, which is a secure online system [44]. Participants will enter their own data online.

### Plans to promote participant retention and complete follow-up {18b}

Participants will be reminded of Death Café debriefing sessions by study investigators on a weekly basis and will receive notifications and reminders for surveys through REDCap. Any participant who fails to complete one or more study surveys or Death Café debriefing sessions will still be invited to complete scheduled follow-up surveys up to the final time point.

Monetary compensation will also be provided for completion of a minimum number of study procedures as follows. For the Death Café intervention arm, participants completing all four surveys and at least 4 Death Café debriefing sessions will receive $140. For the control arm, participants completing all four surveys will receive $40.

### Data management {19}

Data will be collected and stored in REDCap on the Tulane University server. The trial investigators and statistician will have access to the final dataset. All data will be password-protected, and the REDCap database has multiple mechanisms in place to ensure data integrity. Additional data management procedures can be provided by the study investigators upon request.

### Confidentiality {27}

The risk of loss of privacy will be minimized by labeling survey responses and data with only an alphanumerical code (the first three letters of the subject’s mother’s maiden name and the last three digits of their phone number) instead of the participant’s name. The surveys will be sent through REDCap survey software. Names and contact information will be used to generate a list of those enrolled in the study, to send participants surveys, and to let those in the intervention arm know the dates and times of Death Café sessions.

The researchers will discuss the need for keeping the proceedings confidential with the Death Café groups at the beginning and end of each session. This will serve to protect both the subjects participating in the Death Café as well as any patients whose cases are discussed.

The research team will keep the survey responses in the REDCap secure online database. The aggregate data from the surveys will be stored in REDCap and in a passkey-protected folder on a password-protected laptop that only the research team can access. Paper documents such as consent forms will be kept in a locked filing cabinet in the faculty advisor’s locked office. Results of the study will be presented in aggregate with no inclusion of names or identifying information. The raw survey responses and consent forms will be kept up to 1 year after duration of the study and then destroyed.

### Plans for collection, laboratory evaluation, and storage of biological specimens for genetic or molecular analysis in this trial/future use {33}

No biological specimens will be collected as a part of this trial.

## Statistical methods

### Statistical methods for primary and secondary outcomes {20a}

Descriptive statistics will be reported for participant recruitment, study dropout, and engagement with the intervention. Baseline characteristics for each group will be reported. All analyses will be conducted on an intention-to-treat basis.

Unadjusted associations between independent risk factors (i.e., age, gender, and presence of primary or secondary outcome) will be assessed using Student’s *t* test or Wilcoxon rank sum test for continuous variables and a chi-square test for categorical variables. These risk factors and scores will be compared between the intervention and control group as well as analyzed from the beginning to the end of the intervention period (pre-survey compared to 3-month and 6-month scores). Mixed model regression will also be utilized to analyze differences between groups. Qualitative thematic analysis of free text response feedback regarding the intervention will be performed. All analyses will be performed using SAS Enterprise Guide 5.1 (SAS Institute, Inc., Cary, NC).

### Interim analyses {21b}

There will not be a formal stopping rule for the trial. The investigators do not anticipate problems that are detrimental to participants.

### Methods for additional analyses {20b}

Subgroup analyses will be performed by role on the healthcare team and for prespecified questions about personal and work environment wellness. Mixed model regression analysis will be used.

### Methods in analysis to handle protocol non-adherence and any statistical methods to handle missing data {20c}

Protocol non-adherence is unlikely given that this is a pilot study, and the two psychotherapists leading the intervention designed the protocol, which was based on the sessions they have been hosting for 2 years. Additionally, Death Café debriefing sessions will be randomly attended by other study investigators throughout the course of the study to monitor consistency of adherence to the Death Café intervention structure.

Participants will receive reminders to complete surveys and financial incentives for completion in an effort to acquire a complete dataset. All data will be included in the dataset.

In the event of missing data, this will not be an issue given our use of mixed model regression analysis.

### Plans to give access to the full protocol, participant-level data, and statistical code {31c}

The full protocol and datasets used in this study will be available from the corresponding author on reasonable request.

## Oversight and monitoring

### Composition of the coordinating center and trial steering committee {5d}

The coordinating center is directed by the principal investigators (MEB and JD). All investigators will meet at least monthly to discuss project progress and any unanticipated challenges. There is no separate trial steering committee.

### Composition of the data monitoring committee, its role, and reporting structure {21a}

There is no additional data monitoring committee. The study investigators will monitor the survey data in the REDCap database on a regular basis for any issues and will discuss any that arise at the monthly study team meetings. Any issues that are detected by study investigators must be reported to the principal investigators (MEB and JD) immediately, who will then escalate to the Tulane University Institutional Review Board when appropriate.

### Adverse event reporting and harms {22}

No serious adverse events are anticipated to result from the trial or intervention. If any arise, adverse events will be immediately reported to the Tulane University Institutional Review Board.

### Frequency and plans for auditing trial conduct {23}

Monthly study team meetings will be hosted as above to continuously evaluate the trial conduct. The Tulane University Institutional Review Board and funding source (Spirit of Charity Foundation) require annual reports. No other audits will be performed, unless requested by the study sponsor, funding source, or Tulane University Institutional Review Board.

### Plans for communicating important protocol amendments to relevant parties {25}

Any protocol amendments will have to be approved by the Tulane University Institutional Review Board and the hospital-based Research Review Committee. They will also have to be updated on clinicaltrials.gov and communicated to the study funder and sponsor.

### Dissemination plans {31a}

The findings of the trial will be presented at local and national or international scientific meetings. The authors also plan to publish the results of this trial in peer-reviewed journals.

## Discussion

### Implications

Burnout is a substantial problem among ICU clinicians and interventions to prevent or reduce burnout are sparse [5, 15, 29]. Identification of interventions that can prevent burnout in physicians and staff caring for critically ill patients is vital. STOPTHEBURN is the first study to evaluate the efficacy of the hospital-based Death Café to prevent burnout in ICU clinicians. Additionally, the virtual platform by which this study is proposed also provides a novel venue that may increase dissemination to in-need critical care providers dealing with COVID-19 worldwide. If this intervention is feasible and effective in this pilot study, a future, multicenter definitive clinical trial will be undertaken. Reducing burnout in ICU healthcare clinicians has the potential to increase satisfaction/engagement with work, reduce healthcare provider turn over, and improve the quality of care provided to patients. If effective, hospital systems should consider implementing Death Cafés to reduce burnout among ICU clinicians.

### Challenges

While we aim to recruit a sample which represents all ICU employees, participants with burnout, depression, and anxiety symptoms may be difficult to engage in a research study. Feeling already overwhelmed, the employees most at risk may not engage with a time-consuming intervention, which could lead to underestimation of the intervention’s effect. We will do our best to increase subject participation by making participation in the study as convenient as possible using virtual platforms and by providing monetary reimbursement for the time participants spend completing surveys and participating in Death Café sessions.

We recognize that our intervention may have differential impact based on a number of factors. It is possible that burnout may vary at different times of the year with busier times leading to higher rates of burnout. The COVID-19 pandemic may increase the level of burnout and also limit the availability of those most in need to seek help. Certain demographic factors (marital status, having children, etc.), factors related to personal wellness (sleep, history of depression or anxiety, etc.), and those related to work environment (recent conflicts or deaths, etc.) may also play a role. We will not specifically recruit participants based on these factors, as our randomized controlled trial structure will help to account for these and other potential confounders. We will be performing multivariate regression analyses and other subgroup analyses to help us to evaluate these relationships. We hope to identify the employee subpopulations who benefit the most from Death Café debriefing sessions.

The COVID-19 pandemic has limited options for hosting in-person Death Cafés due to the need for social distancing and limiting gatherings to less than 10 participants. As a result, Death Café interventions will be adapted to an online format via a virtual platform such as Zoom. We suspect the virtual modality will make it easier for participants to attend and affiliate outside the workplace environment. Death Cafés are traditionally in-person gatherings, so transition to virtual meetings is a departure from the norm. There is concern that moving to an online platform may lead to a reduction in therapeutic efficacy given that in-person interaction is believed to be a fundamental feature of Death Cafés. Given the need to adapt to virtual social gatherings in the wake of COVID-19, many are now more comfortable with this modality and even expect this platform to serve as an appropriate social milieu for larger gatherings. That said, participation in Death Cafés from home may limit participants’ ability to share stories if they do not perceive strict confidentiality in their home setting (i.e., proximity of family members), and the virtual setting may introduce other confounders (distractions, technological difficulties, etc.). Virtual Death Cafés are arising in the community de novo, and thus, we aim to adapt our intervention to what we see as an emerging trend.

## Trial status

Trial enrollment will begin July 1, 2020, at UMC and is anticipated to conclude July 1, 2021. The current study protocol is dated May 4, 2020.

## Data Availability

The datasets used and/or analyzed during the current study will be available from the corresponding author on reasonable request.

## Abbreviations

GAD-7: Generalized Anxiety Disorder 7-item scale
ICU: Intensive care unit
PHQ-8: Patient Health Questionnaire 8
MBI: Maslach Burnout Inventory
